# The Relationship between the Antitumor Effect of the IL-12 Gene Therapy and the Expression of Th1 Cytokines in an HPV16-Positive Murine Tumor Model

**DOI:** 10.1155/2014/510846

**Published:** 2014-04-07

**Authors:** Flor García Paz, Vicente Madrid Marina, Ausencio Morales Ortega, Abimelec Santander González, Oscar Peralta Zaragoza, Ana Burguete García, Kirvis Torres Poveda, José Moreno, Juan Alcocer González, Eva Hernandez Marquez, Victor Bermúdez Morales

**Affiliations:** ^1^Division Chronic Infection and Cancer, Research Center in Infections Diseases, National Institute of Public Health, Avenida Universidad 655, Col. Santa María Ahuacatitlán, 62100 Cuernavaca, MOR, Mexico; ^2^Institute of Immunology and Virology, School of Biological Sciences, Autonomous University of Nuevo Leon, Avenida Pedro de Alba y Manuel Barragán, 66450 Monterrey, NL, Mexico

## Abstract

*Objective*. The goal of the present study was to investigate the effect of IL-12 expressed in plasmid on the Th1 cytokine profile in an experimental HPV16-positive murine tumor model and the association with the IL-12's antitumor effect. *Methods*. Mice were injected with BMK-16/myc cells to establish HPV16-positive tumor and then pNGVL3-mIL-12 plasmid; pcDNA3 plasmid or PBS was injected directly into tumor site. The antitumor effect of the treatment was evaluated and the cytokines expression profile in each tumor tissue was analyzed. *Results*. Treatment with pNGVL3-mIL-12 plasmid had a significant antitumor effect, and a Th2-Th3-type cytokines prolife was detected in the murine tumor model with expression of the cytokines IL-10, IL-4, and TGF-**β**1. However, after the tumor was treated with three intratumoral injections of plasmid containing IL-12 cDNA, it showed a cytokine profile associated with Th1 with expression of IL-2, IL-12, and IFN-**γ** cytokines and reduced expression of IL-10, IL-4, and TGF-**β**1. *Conclusions*. The treatment with the IL-12 gene in the experimental HPV16-positive tumor model promoted the activation of the cellular immune response via expression of a Th1-type cytokine profile and was associated with the inhibition of tumor growth. Thus, IL-12 treatment represents a novel approach for gene therapy against cervical cancer.

## 1. Introduction

Cervical cancer continues to be a serious public health problem worldwide and there is much room for improvement in the prevention, control, and treatment of this neoplasia. Persistent high-risk human papillomavirus (HPV) infection is the etiologic agent associated with cervical cancer, which together with environmental, social, genetic, and host factors may influence the risk of disease progression [1, 2]. The immune response is a critical factor in regulating tumor growth; however, patients with advanced cancer often exhibit a poorly functioning immune response, which manifests as decreased T cell proliferation, a reduced CD4 : CD8 ratio, alterations in signal transducing molecules (such low CD3*ζ* expression in T cells and expression of MHC class I in the tumor cells), and deficient cellular immune response production of Th1 cytokines, changes which correlate with the severity of the disease [3, 4].

There is substantial evidence that cervical cancer is associated with HPV infection, and that anti-inflammatory and immunosuppressive cytokines are expressed in the cervical microenvironment, determining the persistence of HPV: in this scenario a local immunosuppressive state could accelerate cellular proliferation and tumor growth. The shift Th1 to Th2 cytokine profiles are expressed in cervical tumors, and IL-10 levels are more highly expressed in the cervical epithelium in patients with cervical dysplasias than in normal cervix [5, 6]. The level of IL-10 expression is directly associated with the degree of cervical lesion and correlates with the presence of HPV infection [7, 8]. The balance of expression of Th1/Th2/Th3-type cytokines such as IL-4, IL-10, and TGF-*β*1 in the cervical mucosa, cervical mucus [9], and tumor cells during HPV infection influences the immune status in the local cervical microenvironment and determines the persistence of HPV, progression of dysplasia into invasive cancer, and cervical cancer progression [4–6].

Activation of the immune system to produce an antitumor response depends on the presentation of antigen to T-cells by antigen presenting cells (APC) and the production of cytokines that promote a Th1 cytotoxic response. Several immunotherapeutic agents have been used to activate a Th1 response; among them, the cytokine genes are the most widely studied in cancer gene therapy. Specifically, IL-12 has been shown to induce eradication of experimental tumors, prevent the development of metastases, and elicit long-term antitumor immunity [10–12]. The antitumor effect of IL-12 is associated with the activation of the CTL, Th1-mediated immune response, and NK cells activation [11–15], as well as the impairment of tumor vascularization [16]. While clinical application of the IL-12 protein has been limited by dose-dependent toxicity, the direct intratumoral administration of the IL-12 gene is reliable and reproducible and may limit the need for systemic cytokine administration. Thus, gene therapy with IL-12 holds promises as an alternative immunotherapeutic approach to cervical cancer [17, 18].

In this study, in an HPV16-positive experimental murine tumor model established with the BMK-16/myc cell line, the application of naked cDNA that encodes IL-12 induced inhibition of tumor growth and favored the expression of IL-2, IL-12, and IFN-*γ* and the low-level expression of IL-10, IL-4, and TGF-*β*1 cytokines. These results indicate that treatment with the IL-12 gene in an HPV16-positive the tumor model promotes the activation of the cellular immune response by facilitating a shift of the Th1 cytokine profile.

## 2. Materials and Methods

### 2.1. Cell Lines and Culture

The BMK-16/myc murine cell line was kindly donated by Dr. Sophi Hallez of the Université Libre de Bruxelles, Rhode-Saint-Genèse, Belgium. This cell line was established by the cotransformation of baby of Balb/c kidney cells from newborn mice with the c-myc gene and the HPV16 genome, as previously described [19]. The cell line was grown in a DMEM medium (Invitrogen, Carlsbad, Calif., USA) and supplement with 10% fetal bovine serum, penicillin/streptomycin (50 *μ*g/mL), 2 mM L-glutamine, and 250 ng/mL fungizone at 37°C in CO_2_.

### 2.2. Plasmids

The pNGVL3-mIL-12 plasmid contains the cDNA that encode to p35 and p40 subunits of the murine IL-12 gene expression (mIL-12) separated by an internal ribosomal entrance site and driven by a single CMV promoter (National Gene Vector Laboratory, University of Michigan, Ann Arbor, MI). The pcDNA3 was used as control plasmid. Both plasmids were transformed into* E. coli DH5*α** strain and kanamycin resistant clones were selected for expansion in overnight cultures. The plasmids were purified using the Endofree Plasmid Mega Kit (Quiagen, Gaithersburg. MD) according to the manufacturers guidelines and were then quantified by a NanoDrop Spectrophotometer (Thermo Scientific). The plasmid was stored at −20°C until use.

### 2.3. Establishment and Treatment of the HPV16-Positive Tumor Model

Balb/c female mice, 6–8 weeks of age, were purchased from Charles River Laboratories (Wilminton, Mas., USA) and were injected subcutaneously into the right flank with 5 × 10^5^ BMK-16/myc cells in phosphate-buffered saline (PBS). When the tumor reaches approximately 30 mm^3^ (volume = minor diameter^2^  × major diameter), the pNGVL3-mIL-12 plasmid was injected directly into tumor site at a final concentration of 50 *μ*g in final volume of 20 *μ*L in PBS; pcDNA3 plasmid was used as a control (*n* = 5 animals were used in each group). The treatment was repeated on day 6 and day 12 and the mice were monitored tree times per week for tumor growth over a 28-day period and tumor sizes were measured (in millimeters) with electronic calipers. The results are expressed as average of tumor diameters +/− standard deviation.

### 2.4. Cytokines Gene Expression by Real Time RT-PCR

Mice were sacrificed to obtain IL-12 gene-treated tumor tissue at different time points after treatment. The tissue was digested and homogenized using proteinase K in the reaction buffer (50 mM Tris-HCL pH 7.5/10 mM CaCl_2_) at 37°C. The mix was diluted with 1 mL of TriPure Isolation Reagent (Roche, USA) and the total RNA was obtain by centrifugation in presence of 200 *μ*L chloroform and precipitated with isopropanol at 4°C. Total RNA was dissolved in diethylpyrocarbonate- (DEPC-) water and was quantified in a NanoDrop Spectrophotometer (Thermo Scientific) and 0.5 *μ*g of each sample was used to analyze the integrity of RNA in a 6% urea polyacrylamide gel. The detection and quantification of mRNA cytokines was carried out using ABI Prism 7900HT Sequence Detection System (Applied Biosystems) validated using ABI TaqMan gene expression assays (Applied Biosystems, Foster city, CA). TaqMan One Step PCR Master Mix Reagents Kit (ABI) was used for reverse transcriptase and PCR. The reaction volume was 10 *μ*L including 5 *μ*L of 2x Master Mix, 0.25 *μ*L of 40x MultiScribe and RNase Inhibitor Mix, 0.5 *μ*L of 20x target primer and probe, Nuclease-free water (Ambion, Austin, TX), and 100 ng of RNA sample. The reaction plates (384) were covered with an optical cap and centrifuged briefly to remove bubbles. The thermocycler conditions were as follows: Stage 1: 48°C for 10 min.; Stage 2: 95°C for 10 min; Stage 3: 95°C for 15 sec, repeat 40 cycles, 60°C for 1 min. All assays were carried out in triplicate prepared for each target mRNA and an internal control gene (GAPDH). The real-time RT-PCR amplifications were accomplished using relative quantification analysis and were performed using the comparative 2-^ΔΔCT^ method using peripheral blood mononuclear cells (PBMC) activated with phytohemagglutinin (PHA) for compared the gene expression (Livak & Schmittgen 2001). ABI TaqMan gene expression assays used for specific transcripts were Mm00441724_m1 (TGF-*β*1), Mm00434256_m1 (IL-2), Mm0043170_m1 (IL-12p40), Mm00439616_m1 (IL-10), mM00445259-M1 (IL-4), Mm00801778_m1 (IFN-*γ*), and Mm00446 190_m1 (IL-6). To detect the transcript of CD4 and CD8, we design the primers with the program Universal ProbeLibrary, Assay Design Center (Roche Applied Science); the primer sequence for CD4 antigen was as forward 5′-ACACACCTGTGCAAGAAGCA-3′ and reverse 5′-GCTCTTGTTGGTTGGGAATC-3′ and TaqMan probe 5′-CAGAGGCT-3 (FAM) (probe no. 110). For CD8 antigen, the sequence were forward 5′-CTCACCTGTGCACCCTACC-3′ and reverse 5′-GCTCTTGTTGGTTGGGAATC-3′, and TaqMan probe was 5′-TGCTGTCC-3′ (FAM) (probe no. 56).

### 2.5. Detection of IL-12 and INF-*γ* Expression Levels

Systemic immune response was assessed by measuring plasma IL-12 levels during gene therapy. Blood samples were taken of tumor-bearing mice treated with pNGVL3-mIL-12, pcDNA3, PBS, and the controls. Serum was collected and assayed the IL-12 (p70), INF-*γ* levels using an enzyme-linked immunosorbent assay (ELISA) according to the manufacturer's instructions. ELISA kits were purchased from R&D Systems (Minneapolis, MN).

### 2.6. Statistics

An ANOVA was performed to analyze the variables and measured the expression cytokines genes and CD4/CD8 antigens in the mice treated with pNGVL3-mIL-12, pcDNA3 plasmids, PBS, or controls. The Mann-Whitney test was used to evaluate quantitative differences in cytokine levels in serum of different experimental groups.

## 3. Results

### 3.1. Inhibition of Tumor Growth by IL-12 Gene Transfer

**Figure 1:**
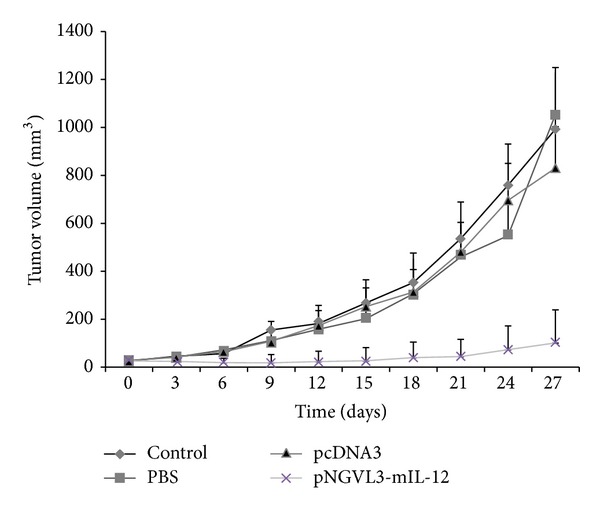
Effect of administration of naked IL-12 DNA on tumor growth. The potential therapeutic effect of naked DNA/IL-12 was evaluated in an experimental tumor model. Each group of mice (*n* = 5) was challenged subcutaneously with 5 × 10^5^ BMK-16/myc cells. When the tumor reached 20–30 mm^3^ in size, in the tumor sites were injected three-times (every six days) with 50 *μ*g pNGVL3-mIL-12 (asterisks), pcDNA3 plasmid (triangle), PBS (square), or no treatment as control (diamond). Tumor size was measured every 3 days over 27 days. Values and bar represent the average and standard deviations of the tumor size. ANOVA, *P* < 0.05.

The antitumor activity of the naked DNA-IL-12 gene was evaluated in an experimental tumor model, generated by subcutaneous injection of the BMK-16/myc cell line into the right flank in syngenic mice as described above. When the tumor reached approximately 30 mm^3^, each mouse was intratumorally injected with 50 *μ*g of the naked plasmid/pNGVL3-mIL-12 and pcDNA3 plasmids, PBS, or not treated. Six and twelve days later of treatment, mice were revaccinated with the same dose and by the same method and tumor growth was monitored for a total of 27 days. The results are shown in Figure 1: inhibition of tumor growth was clearly observed in the mice treated with the pNGVL3-mIL-12 plasmid for at least 15 days, *P* < 0.05. This result correlated with the administration and continued presence of IL-12 DNA in the tumor tissues. The antitumor effect is very evident in all three doses tested; however, after the last administration with the pNGVL3-mIL-12 plasmid, the tumors grew progressively in three of five animals, indicating that the antitumor effect of naked IL-12 DNA is not permanent and depends on the continuous administration of the plasmid into the tumor tissue. In contrast, mice with tumor treated with an irrelevant plasmid (pcDNA3), PBS, or nor treated showed no inhibition of tumor growth. These findings confirm the antitumor effect of IL-12 and the potential use of gene therapy for treatment of cervical cancer. Our challenge in achieving this aim is to develop sustained-release systems to ensure constant expression of the IL-12 and maintain tumor inhibition.

**Figure 2:**
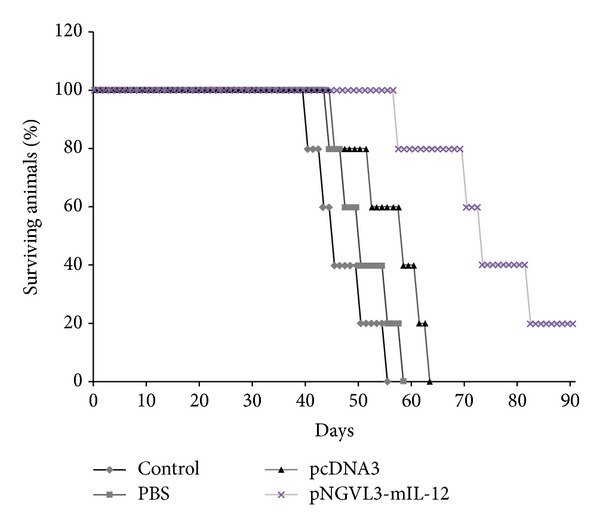
Long-term survival of animal after treatment. The antitumoral effect of IL-12 was evaluated in an HPV16-positive experimental tumor model. The groups of mice (*n* = 5) were injected with plasmid pNGVL3-mIL-12 (asterisks), pcDNA3 plasmid (triangle), PBS (square), or no treatment as control (diamond). Mouse survival was evaluated each day after the treatment, increased survival of tumor-free mice treated with IL-12 compared with controls.

In addition to determining the antitumor effect of IL-12, we monitored mouse survival and reported the percentage living treated and sham-treated animals versus time from initiation of treatment. As shown in Figure 2, mice that received pNGVL3-mIL-12 DNA vaccination had a survival rate of approximately 60% after the treatment relative to tumor-free mice and had higher survival than mice treated with pcDNA3 plasmid, PBS, or not treated. Although treatment with pNGVL3-mIL-12 plasmid does not generate a complete inhibition of tumor, it does retard tumor growth and causes an increase in mouse survival. Our data confirm the antitumor properties of IL-12 in the HPV16-positive tumor model and are consistent with previous reports that used pNGVL3-mIL-12 plasmid in murine CT26 liver tumors and in murine mammary adenocarcinoma and renal cell carcinoma models [20]. These findings confirm the antitumor effect of IL-12 on inhibition of sustained tumoral mass growth in this biological tumor model and shed light on the potential use of gene therapy for treatment of cervical cancer.

### 3.2. Cytokine Expression Profiles of in the Tumor after Treatment with IL-12 DNA

**Figure 3:**
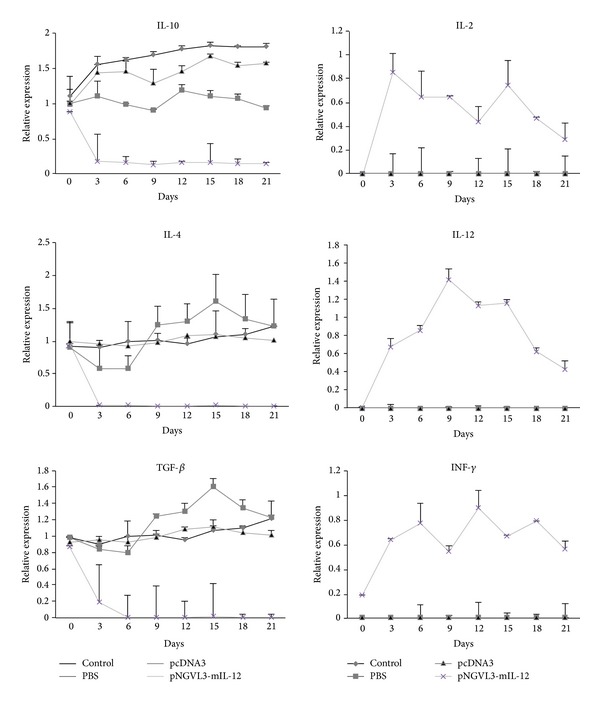
Intratumoral expression levels of cytokines mRNA. An experimental tumor model was generated and treated with the IL-12 gene expression plasmid (asterisks), pcDNA3 plasmid (triangle), PBS (square), or no treatment as control (diamond). Every three days following treatment the mice were sacrificed, the total RNA was extracted, and the cytokines expression profile was determined with by real time RT-PCR. Th2-Th3-type cytokine profiles and no expression of Th1-tye cytokines were detected in the tumor-bearing mice prior to the treatment. A significantly lower expression of Th2-/Th3 cytokines and higher expression of Th1 cytokines were detected after the treatment with IL-12. Results represent the means ± S.E. values of cytokines expression in each 5-mouse group, *P* < 0.001.

The antitumor activity of IL-12 has been linked to its ability to target multiple cell populations including T cells and NK cells and to induce IFN-*γ* and activate cytokines. However, little is known about the influence of IL-12 on the cytokine profile expressed by tumor tissues and by cells in the tumor microenvironment. We analyzed the cytokine profiles prior to and during IL-12 gene therapy in the HPV16-positive murine tumor model. Detection of mRNA levels of several cytokines in the mice was accomplished by real time RT-PCR and the analysis was performed by comparing the cytokines expressed by murine peripheral blood mononuclear cell activated with PHA (control) because some cytokines are not expressed by the tumor tissue. As seen in Figure 3, expression of IL-10, IL-4, and TGF-*β*1 cytokines was detectable in the tumor tissue (pretreatment); we previously demonstrated that these cytokines are expressed by the BMK-16/myc cell line in Supplementary Material available online at http://dx.doi.org/ 10.1155/2014/510846, which suggests that IL-10, IL-4, and TGF-*β*1 may promote establishment of the tumor in mice. Furthermore, the levels of these cytokines show a tendency to increase their expression according to tumor growth and found no significant differences in the control mice, and mice treated with PBS and pcDNA3 plasmid. The predominant expression of cytokines with immunosuppressive properties such as IL-10 and TGF-*β*1 could downregulate the cellular immune response favoring tumor implantation and promoting tumor growth in mice.

**Figure 4:**
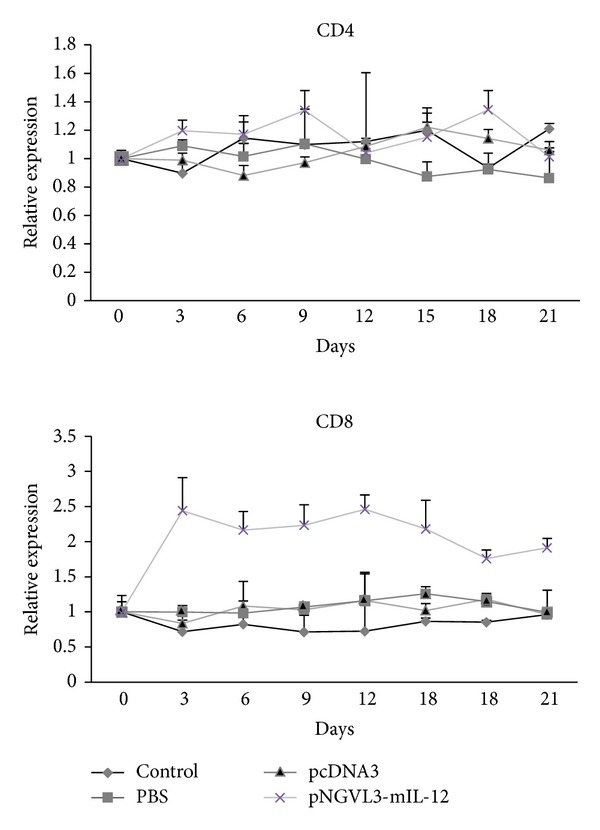
Intratumoral expression levels of CD4 and CD8 mRNA. Tumor-bearing mice were treated with the IL-12 gene expression plasmid (asterisks), pcDNA3 plasmid (triangle), PBS (square), or no treatment as control (diamond). Every three days following treatment the mice were sacrificed and by real time RT-PCR the CD4 and CD8 mRNA were detected. The CD8 mRNA level expression was higher than the CD4, however, not significant were detected. Results represent the means ± S.E. values of in each 5-mouse group, *P* > 0.01.

In the tumor-bearing mice that were treated with the pNGVL3-mIL-12 plasmid, the intratumoral cytokine expression profile was affected, showing a significant decrease in expression of IL-4, IL-10, and TGF-*β*1 (Th2/Th3-type cytokines) (*P* value < 0.001) after the first administration of plasmid, which correlated with inhibition of tumor growth. The expression of these cytokines is maintained at a low level during the IL-12 plasmid treatment, but IL-10 expression is not completely inhibited. Presistence of pNGVL3-mIL-12 plasmid in the tumor microenvironment can explain the partial inhibition of IL-10 expression and the therapeutic effect on tumor growth at 21 days. On the other hand, the injection of IL-12 cDNA led to a significantly increased expression of IL-2, IL-12, and IFN-*γ* (Th1-cytokines) (*P* value < 0.001). Of note, the Th1 cytokines were not detected in the tumor tissue without treatment with IL-12 plasmid in the BMK-16/myc cell line. The IL-12 expression was higher than IL-2 and IFN-*γ*, which is due to administration of the pNGVL3-mIL-12 plasmid. Furthermore, mRNA expression levels of Th1 cytokines were high after IL-12 gene therapy and correlated with tumor inhibition in the mice. However, it is necessary to consider that the detection of the expression of IL-12 in tumor-bearing mice treated with the plasmid pNGLV3-mIL-12 can be influenced by the expression plasmid produced by itself or by the infiltrating tumor cells. We paid special attention to the differences in the Th1, Th2, and Th3 cytokines during treatment with IL-12. The shift from Th2-Th3 cytokines to Th1 cytokines suggests that IL-12 gene transfer in the tumor model promotes the cellular immune response and favors differentiation to Th1 among the tumor infiltrating cells [21]. The Th1 cytokine profile may induce effector cells as CD8+ T cells and NK-T cells that can function to eliminate tumor cells and promote tumor regression. We found that the CD4 and CD8 mRNA expression levels of T cells in mice treated with IL-12 gene therapy increased relative to the control, and the CD8 mRNA level expression was higher than the CD4 (Figure 4) which correlates with previous reports that IL-12 mediated tumor regression and is dependent on CD8+ T cells [22].

### 3.3. Serum Levels of IL-12 (p70) and IFN-*γ* Cytokine

**Figure 5:**
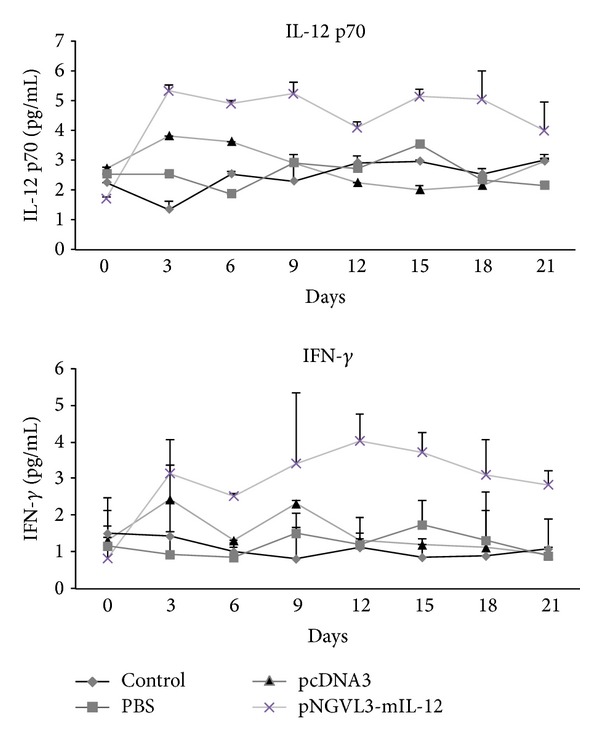
Levels of IL-12 (p-70) and IFN-*γ*. Tumor-bearing mice were treated with the IL-12 gene expression plasmid (asterisks), pcDNA3 plasmid (triangle), PBS (square), or no treatment as control (diamond). Every three days following treatment, the serum was collected and by ELISA were detected the IL-12 p70 and IFN-*γ* proteins. Results represent the means ± S.E. values (error bars) of in each 5-mouse group, *P* > 0.05.

To determine whether the antitumor effect of naked DNA-IL-12 gene in HPV 16 experimental murine tumor is related to systemic levels of the IL-12 protein, we measured serum levels of IL-12 by ELISA and found very discrete increases in the expression of this cytokine were detected in the serum of mice treated with IL-12 gene (4.22 pg/mL) in comparison with control mice (2.40 pg/mL control, 2.52 pg/mL PBS, and 2.72 pg/mL pcDNA3), not statistically significant (*P* > 0.05 Mann Whitney-*U*). In addition we did not found a significant increase compared with the levels of the control mice (Figure 5). However, we believed that this small increase of IL-12 and IFN-*γ* is enough to reduce the tumor size in this mice model.

## 4. Discussion

IL-12 is a potent cytokine with a central role in regulating innate and adaptive immune responses [13]. It is produced by activated macrophages, dendritic cells, and B cells and is involved in the regulation of NK cells, peripheral blood mononuclear cells, and B cells; it is also required for optimal differentiation of CD4+ T cells into type 1 T helper cells (Th1) and promotes cell-mediated immunity [12, 13]. The antitumor, antimetastatic, and antiangiogenic activities of IL-12 have been demonstrated in many murine tumor models and it has been used in clinical trials for the treatment of certain human cancers [23]. IL-12 is a promising cytokine for cancer treatment, but extensive use in the clinic has been limited by severe toxicities, including death, that prevent its systemic administration to reach therapeutic levels in solid tumor lesions in cancer patients [24, 25]. Alternative approaches for IL-12 delivery such as gene therapy and gene-modified tumor cell vaccines, which have been developed for the sustained local delivery of cytokines in cancer, could facilitate the clinical applications of IL-12.

IL-12 gene immunization may be an effective therapeutic strategy against cervical cancer and could be applied locally to promote the cellular immune response. Previously, we reported that IL-10 and TGF-*β*1 are expressed in cervical lesions; therefore, the development of SIL and cervical cancer is preferentially associated with the expression of immunosuppressive cytokines (IL-10 and TGF-*β*1) and Th2-type cytokines (IL-4/IL-6) [26]. HPV+ tumor cells themselves express IL-10 and TGF-*β*1 and this modulates tumor cell proliferation, affects the local immune response, and generates an immunosuppressive state. Our goal in the present study were to generate an HPV16-positive experimental tumor model with a Th2 cytokine expression profile to evaluate the IL-12 gene as an activator of the cellular immune response and to explore its antitumor effect as a potential cervical cancer treatment. Additionally, we analyzed the intratumoral cytokine expression profile prior to and in response to treatment with the IL-12 gene in the HPV16-positive experimental tumor model.

In this study, we used the BMK-16/myc murine cell line transformed with HPV16 to establish murine HPV tumor model. Previously, we determined that the cell line expresses IL-4, IL-10, and TGF-*β*1 cytokines indicators of a Th2 and Th3-type profile. Theses cytokines are similar to the profile expressed in human cervical cancer tissues and in squamous cell cervical carcinoma [5, 27]. The cytokine profile expressed by the BMK-16/myc cells may promote cell proliferation and cell implantation in syngenic H2^d^ mice Balb/c to generate the tumor model. We observed a high percentage of tumor-bearing mice (90%) following administration of the BMK-16/myc cells over a period of 4-5 days: significantly experimental tumor growth is constant and lethal for the mice. The tumor model was generated in immunocompetent mice and it may be that the immunosuppressive cytokines expressed by the BMK-16/myc cells negatively influence the mouse immune response and facilitate implantation of tumor cells. Therefore, this tumor model provides a means to study interactions between the immune system and tumors and therapies that target such interactions. To this end, we evaluated the expression profile of cytokines Th1/Th2/Th3 cytokines in the tumor model generated. In the tumor tissues biopsies analysed, IL-4, IL-10, and TGF-*β*1 were the main cytokines expressed and their expression was maintained during growth of the tumor. As previously mentioned, these cytokines are constitutively expressed by the BMK-16/myc cells and support this application of cell implantation in Balb/c mice to generate tumor. The Th2-Th3-type cytokine profile detected in the murine tumor model correlates with studies, which have demonstrated a similar cytokine expression profile in biopsies of cervical cancer and fluids from the cervix and vagina [27–29]. These findings suggest that this cytokine expression profile has a special role in multiple immunological regulatory events that promote a local immunosuppression state in mice and favor the tumor implantation during tumorogenesis.

The evaluation of the antitumor effect of the IL-12 gene in the tumor model generated was evaluated in groups of tumor-bearing mice. Mice treated with the IL-12 gene showed a clear inhibition of tumor growth for at least 15 days, by contrast tumor-bearing mice treated with an irrelevant plasmid (pcDNA3), PBS, or not treated, did no show any inhibition of tumor growth, with significant difference (*P* < 0.05). However, tumor inhibition by the IL-12 gene is dependent on administration and permanence of naked DNA in the tumor tissues. The antitumor effect was evident for all three doses tested (administered every six days); however after the final administration of pNGVL3-mIL-12 plasmid, the tumors grew progressively in three of five animals, indicating that the antitumor effect of naked DNA IL-12 gene is not permanent and depends on the continuous administration of the plasmid into the tumor tissue. It should be noted that the antitumor effect of IL-12 has been observed in several tumor models [11, 12, 23, 25] and been shown to prevent metastases, inhibit angiogenesis, and activate the cellular immune response. These findings confirm the antitumor function of IL-12 and its potential use in gene therapy for the treatment of cervical cancer.

The antitumor effect of the IL-12 gene in several tumor models has been linked to activation of the cellular immune response, mainly activation of CD4+ T cells and CD8+ T cells [21]. Previously, we detected CD4 and CD8 mRNA in biopsies of tumor tissues from tumor models treated with the IL-12 gene. Remarkably high levels of CD8 molecule were detected in the tumor-bearing mice treated with IL-12 and help explain the antitumor effects of IL-12. Furthermore, we demonstrate that gene therapy using the IL-12 cytokine in the experimental tumor model in immunocompetent HPV16-positive mice induces a shift from Th2-Th3 to a Th1-type cytokine expression profile, which is associated with tumor regression (Figure 3). However, the antitumor effect of IL-12 and its expression is reflected only in the tumor microenvironment; very discrete changes of IL-12 and IFN-*γ* were detected in the serum of mice treated. The ability of IL-12 to promote to the Th1-type cytokines expression profile had been reported in other tumor models [30]. Nevertheless, it was not clear whether IL-12 mediated inhibition of expression of Th2 and Th3-type cytokines in tumor models, but it has been observed that IL-12 reduces the immunosuppressive microenvironment of tumor and eradicates colorectal cancer metastases in mice [24].

Finally, IL-12 has become one of the most effective antitumor cytokines in experimental animal models and it has been demonstrated that the IL-12 gene therapy has far fewer toxic side effects that administration of the IL-12 protein. Preclinical trial studies have shown that the intratumoral application of IL-12 gene therapy can induce a striking antitumor response in several murine tumor models including melanoma, sarcoma [25], ovarian [12], breast, colorectal [24], lung [30], laryngeal [31], and in cervical cancer [32, 33]. In conclusion, we propose that based on data reported here, IL-12 can be considered an excellent cytokine candidate for the treatment of low- and high-grade cervical lesions associated with HPV infection.

## 5. Conclusions

In the present study we detected a Th2/Th3-type cytokine expression profile (IL-10, IL-4 and TGF-*β*1) in an HPV16-positive murine tumor model and found that this profile changes following treatment with the IL-12 gene. We determined a clear antitumor effect of IL-12 plasmid DNA against an experimental murine tumor, which was associated with shift to a Th1-type cytokines profile, with expression of IL-2, IL-12 and IFN-*γ* cytokines and reduced expression of IL-10, IL-4 and TGF-*β*1 cytokines. Together, these results suggest that the treatment with the IL-12 gene in the tumor model promotes activation of the cellular immune response against an HPV-positive tumor via expression of Th1-type cytokines profile and promotes inhibition of tumor grown. IL-12 likely represents an additional approach for gene therapy against cervical cancer.

## Supplementary Material

Detection of cytokines expressed by BMK-16/myc cells. Five-microgram of total RNA extracted from BMK-16/myc cells were reversed transcribed and amplified by PCR. RT-PCR was performed, using specific primers for IFN-*γ*, IL-6, IL-4, TNF-*α* and TGF-*β*. PCR products were DNA were electrophoresed through 6% acrylamide gels and visualized with ethidium bromide staining. We used murine peripheral blood mononuclear cell activated with PHA as cytokines control. We detected that the BMK-16/myc cells expressed IL-4, TNF-*α* and TGF-*β*.
