# Supplementary figures and images for: The Relationship between the Antitumor Effect of the IL-12 Gene Therapy and the Expression of Th1 Cytokines in an HPV16-Positive Murine Tumor Model
Source: Mediators Inflamm. 2014 Apr 7;2014:510846. doi: 10.1155/2014/510846 (PMC3997981; doi:10.1155/2014/510846)

# Detection of cytokines in BMK-16/myc cell by RT-PCR

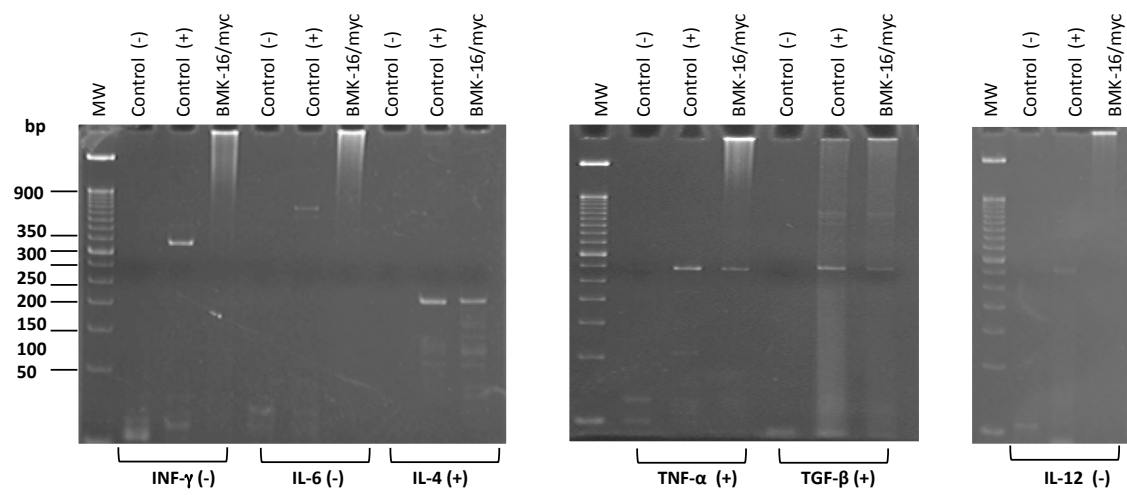

Supplementary material

Supplement: Supplementary file 1 — Detection of cytokines expressed by BMK-16/myc cells. Five-microgram of total RNA extracted from BMK-16/myc cells were reversed transcribed and amplified by PCR. RT-PCR was performed, using specific primers for IFN-γ, IL-6, IL-4, TNF-α and TGF-β. PCR products were DNA were electrophoresed through 6% acrylamide gels and visualized with ethidium bromide staining. We used murine peripheral blood mononuclear cell activated with PHA as cytokines control. We detected that the BMK-16/myc cells expressed IL-4, TNF-α and TGF-β. [file 510846.f1.pdf]
